# Muscle tissue engineering and regeneration through epigenetic reprogramming and scaffold manipulation

**DOI:** 10.1038/srep16333

**Published:** 2015-11-09

**Authors:** S.J. Tan, J.Y. Fang, Y. Wu, Z. Yang, G. Liang, B. Han

**Affiliations:** 1 Department of Biomedical Engineering, Viterbi School of Engineering, University of Southern California, Los Angeles, CA; 2 Nimni-Cordoba Tissue Engineering and Drug Discovery Laboratory, Division of Plastic and Reconstructive Surgery, Department of Surgery, Keck Medical School, University of Southern California, Los Angeles, CA; 3 Department of Urology, Keck School of Medicine, University of Southern California, Los Angeles, CA 90089, CA

## Abstract

Efficiency of cell-based tissue engineering and regenerative medicine has been limited by inadequate cellular responses to injury because of aging and poor controllability of cellular interactions. Since cell progression is under a tight epigenetic regulation, epigenetic modulators such as 5-azacytidine (5-Aza-CR) have been utilized to facilitate reprogramming and development of somatic cells in 2-dimensional (2-D) settings. Nonetheless, progression of a specific tissue lineage toward the terminal phenotype is dependent not only on the genomic potential, but also on the microenvironment cues that are beyond the capability of 2-D approaches. In this study, we investigated the combined effects of matrices of variable rigidities and the treatment with the epigenetic modulator 5-Aza-CR on reprogramming adipose-derived stromal cells (ADSCs) into myoblast-like cells by utilizing tunable transglutaminase cross-linked gelatin (Col-Tgel) *in vitro* and *in vivo*. Our experiments demonstrated that cellular plasticity and trans-differentiation were significantly enhanced when ADSCs were treated with an effective dose of 5-Aza-CR (1.25 to 12.5 ng) in the optimal myogenic matrix (15 ± 5 kPa Col-Tgel). Our findings suggest that both physical signals and chemical milieu are critical for the regulation of cellular responses.

Regeneration of extensive skeletal muscle damage resulting from aging, trauma, or tumors, and restoration of its normal structure and function remain daunting challenges for regenerative medicine^1^. Even though cell-based tissue engineering may be one of the promising strategies for tissue repair, efficacy of transplanted cells in the adult body as well as the spatial and temporal precision of their interaction with the extracellular matrix (ECM) remain ambiguous^2^,^3^. The use of cells derived from adult tissues is favored over the application of embryonic stem cells (ESCs), as practical utility of ESCs is limited by problems resulting from cell regulation and ethical considerations. Furthermore, a decreased immunogenicity^4^ and potential “immunosuppressive” properties that have been described in various adult cells^5^ may facilitate allogeneic transplantation, providing further advantages of these cells for regenerative medicine. On the other hand, some studies have emphasized the inadequacy of the response of adult stem cells to disease and injury due to cell maturation associated with a gradual loss of their differentiative potency^4^,^5^. Common protocols for cell-based therapeutics include such steps as tissue harvesting, isolation and expansion of cells, their storage, and subsequent transplantation to patients. These steps introduce functional changes in cells, which may dramatically affect their plasticity and therapeutic utility.

Nonetheless, the development of nuclear cloning^6^ and induced pluripotent stem cells (iPSs)^7^ has highlighted the possibility to reverse the fate of a committed or differentiated cell by epigenetic reprogramming. DNA methylation is one of the most important epigenetic events and is frequently described as a ‘silencing’ epigenetic mark^8^,^9^. It plays a major role in regulating the production of distinct cell types by adding methyl groups to cytosine nucleotides in DNA and recruiting histone deacetylases (HDACs) that promote condensation of DNA around histones, thereby silencing gene expression. Inhibitors of DNA methylation stimulate the removal of methyl groups from DNA nucleotides in a process known as DNA demethylation, rapidly reactivating the expression of genes that have undergone epigenetic silencing and switching on silenced pluripotency genes. Prototype inhibitors of DNA methylation, such as 5-azacytidine (5-Aza-CR), were initially developed as cytotoxic agents^10^, but it was subsequently discovered that they powerfully suppress DNA methylation and induce gene expression and differentiation in cultured cells^11^,^12^. Thus, it should be possible to produce pluripotent stem cells from adult tissues by inducing DNA demethylation with epigenetic modulators, thereby bypassing complications of cloning or iPSC generation procedures.

There have been numerous attempts^12^,^13^,^14^ to utilize epigenetic drugs in order to promote the development of somatic cells by cellular reprogramming using chemical signals in two-dimensional (2-D) settings. However, several studies^15^,^16^ have provided overwhelming evidence that the progression of specific tissue lineage toward the final phenotype depends not only on the genomic potential of the cells, but also on local environmental cues. These environmental factors, which include mechanical restriction, oxygen concentration, and cell-matrix interactions, cannot be adequately accounted by 2-D models. Furthermore, cell and environmental interactions are omnidirectional, dynamic, and complex in nature, whereas cellular interactions in 2-D are only bidirectional^17^. Therefore, a three-dimensional (3-D) scaffold that mimics the physiological microenvironment is of paramount importance for studies of the aforementioned physiological events, as well as for providing a spatially and temporally template for complex multicellular processes of tissue formation and regeneration *in vitro* and *in vivo*^18^,^19^. Most importantly, 3-D scaffold naturally assumes the role of a carrier that delivers transplanted cells and/or chemical stimuli^18^,^20^. Besides, it is able to provide an adequate support at the injury site and induce *in situ* ingrowth and differentiation of cells from healthy residual tissues, especially in conditions accompanied by extensive losses of the full-thickness native tissue architecture.

In our previous studies of 3-D scaffolds, we demonstrated the suitability of tunable transglutaminase cross-linked gelatin (Col-Tgel) for *in vitro* experiments as well as *in vivo* delivery and repair^18^,^21^. A particularly useful feature of Col-Tgel is its mechanical strength that supports cell adhesion, survival, and organization during the regeneration process without compromising effects of bioactive substances. Moreover, matrix stiffness of Col-Tgel can be conveniently tuned by controlling the concentration of gelatin providing means of fine regulation of cellular responses. Because of these beneficial properties, Col-Tgel was utilized in this study to examine the capability of the epigenetic modulator 5-Aza-CR to reprogram adipose-derived stromal cells (ADSCs) into myoblasts-like cells in both *in vitro* and *in vivo* models. ADSCs were chosen because of the simple surgical manipulation involved, a possibility of easy and repeated access to the abundant subcutaneous adipose tissue, and straightforward enzyme-based isolation procedures. Since effects of microenvironmental changes and their interactions with epigenetic drugs have yet to be adequately explored, the interaction effects of 5-Aza-CR and the microenvironment on cellular responses including activation and/or deactivation of gene(s), cellular remodeling, and self-regenerate capability were evaluated to provide new insights into cell reprogramming, development and maturation, as well as material-cell-based regeneration.

## Results

2-D studies *in vitro*^22^,^23^ have shown that brief exposures to low doses of epigenetic modulators are sufficient to result in 85%–90% alterations of the epigenetic genome; however, cells cultured in 2-D may not have the same functional characteristics as in the 3-D physiological environment. Thus, a number of more physiologically relevant 3-D *in vitro* studies were performed by utilizing the Soft (0.9 ± 0.1 kPa), Med (15 ± 5 kPa), and Stiff (40 ± 10 kPa) Col-Tgels. We observed that an intermediate dose of 5-Aza-CR (*i.e.*, 1.25–12.5 ng) had the maximum effect on ADSCs to trans-differentiate into myoblasts-like cells, as depicted in Fig. S1A. Low (*i.e.*, < 0.125 ng) and high (*i.e.*, > 67.5 ng) doses of 5-Aza-CR examined did not significantly enhance the trans-differentiation of ADSCs into myoblast-like cells. Moreover, a high dose of 5-Aza-CR programmed the treated cells to undergo apoptosis, as shown by the caspase 3/7 staining method (Fig. S1B). Hence, the intermediate dose of 5-Aza-CR was used throughout this study.

### Loss of ADSCs original phenotypes

**Figure 1:**
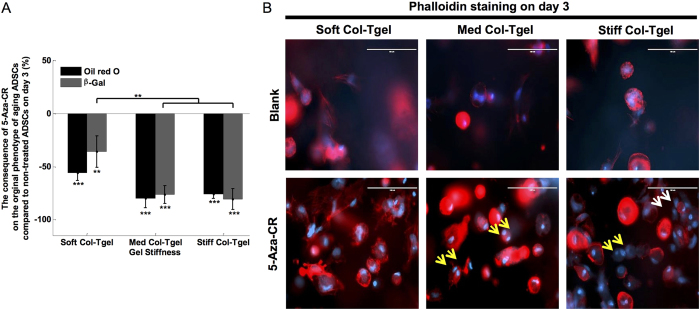
Loss of unique cellular identities by ADSCs. ADSCs were encapsulated in the Soft, Med, or Stiff Col-Tgel and grown with or without 5-Aza-CR. (**A**) Oil red O colorimetric assay and β-gal staining. The Oil red O assay and β-gal staining revealed a downregulation of the original phenotype of aging ADSCs in the presence of 5-Aza-CR in all gel types. However, a more pronounced effect was observed in stiffer gels (*i.e.*, Med and Stiff Col-Tgels) compared to the Soft Col-Tgel. Values in the treated group were normalized by corresponding values in the non-treated group and scaled between 100% (upregulation) and −100% (downregulation). Data are presented as the mean ± standard deviation of 4 samples and statistical differences (Tukey-Kramer HSD test) are indicated as follows: *P < 0.05, **P < 0.01, and ***P < 0.001. (**B**) Phalloidin staining. Cells embedded in the Soft Col-Tgel appeared mesh-like or exhibited extended actin filaments, whereas cells that grew in the Med and Stiff Col-Tgels had dot-like actin microfilaments and filopodia. Cells treated with 5-Aza-CR became larger and more spherical, particularly in stiffer matrices. Yellow and white arrows in the figure indicate representative multinucleated and cell clusters, respectively. The multinucleated and cluster cells frequently appeared in stiffer matrices treated with 5-Aza-CR, but they were rarely observed in the soft platform (Scale bars, 100 μm).

The uniform staining of the ADSCs and aging markers in combination with the lack of expression of pluripotency and myogenic markers demonstrated that ADSCs encapsulated in the Soft, Med, and Stiff Col-Tgels were initially not myoblast-like or stem-like cells (Fig. S2). Oil Red O staining and colorimetric assays were performed to visualize and quantify adipocytes, respectively^24^. As a consequence of demethylation induced by 5-Aza-CR in 3-D, the absorbance of Oil red O revealed by the Oil red O assay was greatly reduced in all gel conditions (Fig. 1A, *P* < 0.001). ADSCs were isolated from aged rats and cells gradually expressed beta-galactosidase (β-Gal) with serial passages in cell culture^25^. Treatment with 5-Aza-CR significantly reduced the number of β-Gal stained aged ADSCs in all gel conditions (Fig. 1A, *P* <0.05). Reprogramming of ADSCs original phenotypes may also be observed through the changes in cell shape and cytoskeleton integrity, since several studies have shown that cell shape regulates cell fate commitment^26^,^27^,^28^. We performed phalloidin staining to visualize the organization of actin filaments (Fig. 1B) and revealed that untreated cells in the Soft Col-Tgel exhibited mesh-like or extended actin filaments, whereas dot-like actin-microfilaments and filopodia were found in cells encapsulated in the Med or Stiff Col-Tgels. Although the cytoskeleton of ADSCs embedded in Col-Tgels and treated with 5-Aza-CR remained similar to that of untreated cells, treated ADSCs became considerably larger and more spherical in all gel conditions. Multinucleated and cell clusters were regularly observed among 5-Aza-CR-treated ADSCs that were encapsulated in stiffer matrices, as shown in Fig. 1B (indicated by yellow and white arrows, respectively). Thus, our results suggested that cell reprogramming and development are not only related to epigenetic events but are also highly dependent on the microenvironment.

### Activation of silent gene(s)

**Figure 2:**
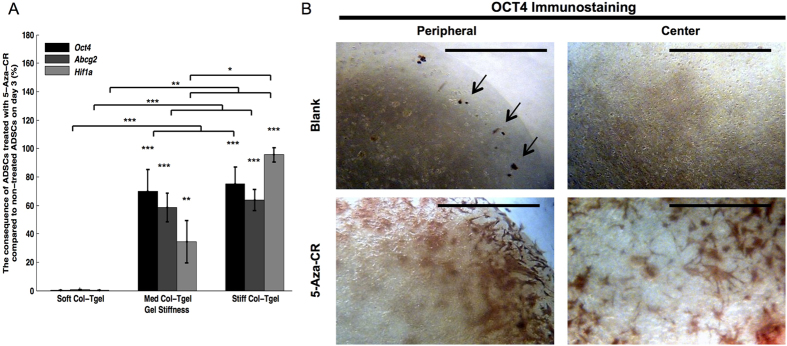
Activation of silent genes. ADSCs were encapsulated in the Soft, Med, or Stiff Col-Tgel and grown with or without 5-Aza-CR. (**A**) Expression of *Oct4*, *Abcg2*, and *Hif1a* was evaluated by RT-PCR. The presence of 5-Aza-CR enhanced the expression of *Oct4*, *Abcg2*, and *Hif1a*. The stem cell markers *Oct4* and *Abcg2*, as well as the hypoxic marker *Hif1a* were highly expressed in stiffer matrices (*i.e.*, Med and Stiff Col-Tgels), whereas much lower expression was observed in the Soft Col-Tgel. Values in the treated group were normalized by corresponding values in the non-treated group and scaled between 100% (upregulation) and −100% (downregulation). Data are presented as the mean ± standard deviation of 4 samples and statistical differences (Tukey-Kramer HSD test) are indicated as follows: *P < 0.05, **P < 0.01, and ***P < 0.001. (**B**) OCT4 immunostaining. Untreated cells were readily activated around the periphery but not within the center of the gel. In contrast, cells grown in the presence of 5-Aza-CR were activated evenly throughout the gel. Black arrows indicate positive staining (Scale bars, 1000 μm).

DNA demethylation is considered as a gene ‘activation’ mark, regulating the “ON” switch of silenced embryonic genes^12^,^14^. Thus, morphological changes were accompanied by the upregulation of the pluripotency expression that was assessed by RT-PCR and immunostaining (Fig. 2A and S3, respectively). The expression of octamer-binding transcription factor 4 (*Oct4*) required to maintain the pluripotency and self-renewal of embryonic stem cells^29^,^30^, was examined. We observed elevated expression of this cell pluripotency marker, indicating that 5-Aza-CR enhanced cellular plasticity in all gel conditions. Yet, the expression of *Oct4* in cells grown in stiffer matrices (*i.e.*, Med and Stiff Col-Tgels) was approximately 80% higher than in cells grown in the soft platform (*P* < 0.001). This observation revealed that the microenvironment plays a vital role in regulating the cellular expression of genes and proteins. In addition to the activation of a stem cell marker, incubation with 5-Aza-CR stimulated the expression of several vital upstream factors including ATP-binding cassette sub-family G member 2 (*Abcg2*)^31^,^32^ and hypoxia inducible factor-1alpha (*Hif1a*)^33^, which mediate a broad range of downstream cellular and systemic responses. As in the case of *Oct4*, the expression of *Abcg2* in cells grown in stiffer matrices (*i.e.*, Med and Stiff) was approximately 70% higher than in cells in the soft platform (*P* < 0.001). 5-Aza-CR stimulated *Hif1a* expression in all gel conditions. Nonetheless, the enhancement was more pronounced with the increase in gel stiffness irrespectively of the concentration of 5-Aza-CR: the highest amount of the *Hif1a* marker was expressed in cells cultured in the Stiff Col-Tgel, whereas much less *Hif1a* signal was present in cells grown in the Soft Col-Tgel. Thus, these results demonstrate that 5-Aza-CR caused differential activation of silent genes depending on the microenvironment.

### Cellular activation

**Figure 3:**
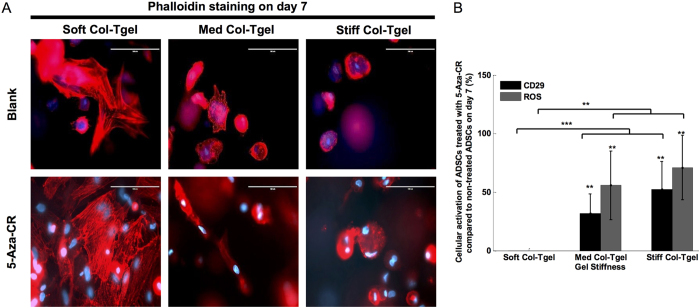
Cellular activation. ADSCs were encapsulated in the Soft, Med, and Stiff Col-Tgels and grown with or without 5-Aza-CR. (**A**) Phalloidin staining. Actin filaments of cells grown in the Soft Col-Tgel were mostly in a mesh-like arrangement or aligned parallel to the directions of elongation and branching. In contrast, dot-like actin filaments and filopodia were frequently found in the Stiff Col-Tgel. The presence of 5-Aza-CR improved the visibility of cell shape and actin cytoskeleton within the same type of gel (Scale bars, 100 μm). (**B**) Expression of Integrin-β1 (CD29) and reactive oxygen species (ROS) production. 5-Aza-CR improved cell-matrix interactions and ROS production, especially those grown in stiffer matrices (Med and Stiff Col-Tgel). Values in the treated group were normalized by corresponding values in the non-treated group and scaled between 100% (upregulation) and −100% (downregulation). Data are presented as the mean ± standard deviation of 4 samples and statistical differences (Tukey-Kramer HSD test) are indicated as follows: *P < 0.05, **P < 0.01, and ***P < 0.001.

Activation of silent genes was observed in the center of gels in cells that were treated with 5-Aza-CR, especially those grown in the Stiff Col-Tgel (Fig. 2B). This observation was contrary to what was observed in untreated cells. Thus, we hypothesized that addition of 5-Aza-CR may increase the sensitivity of cells toward the microenvironment. Previous studies have shown that focal adhesions could be used as force sensors since cells interact with the matrix through anchored actin-microfilament bundles^34^,^35^. Therefore, on day 7, phalloidin and Integrin-β1 (CD29) staining were performed to visualize the organization of actin filaments (Fig. 3A) and to assess the degree of cell-matrix interactions, respectively (Fig. 3B). Actin filaments of cells in the Soft Col-Tgel either formed a mesh-like arrangement or aligned parallel to the direction of elongation and branching. In contrast, formations of dot-like actin filaments and filopodia were frequently observed in cells cultured in the Stiff Col-Tgel. Actin filaments of ADSCs encapsulated in the Med Col-Tgel appeared either aligned parallel to the direction of elongation or formed dot-like filopodia. Addition of 5-Aza-CR did not change the cell shape and actin organization in any gel, but enhanced the already present phenotypes in all gel types investigated. Therefore, differences in cell shape and actin organization in cells cultured on different gels became particularly pronounced, indicating that ECM tends to control the differentiation destiny of cells. Positive CD29 staining further revealed that micron-sized cortical protrusions of cells entered the surrounding matrix. The number of protrusions was found to be higher in ADSCs treated with 5-Aza-CR compared to untreated ADSCs in all gel conditions. Notably, the protrusions were rare in the Soft Col-Tgel, but were quite abundant in the Stiff Col-Tgel (*P* < 0.001). Data on CD29 immunostaining suggest that the addition of 5-Aza-CR did not alter the tendency of cells to interact preferentially with a stiff microenvironment compared to a softer platform. These results also indicate that 5-Aza-CR enhanced the sensitivity of the ADSCs respond to the tension exerted by the microenvironment. In addition to signaling, the underlying metabolism of cellular states is likely to change during the transition from quiescence to activation^36^. Thus, reactive oxygen species (ROS) staining was performed to assess the level of ROS production in different environments (Fig. 3B and S4A). Figure S4 displayed that the highest ROS expression was found in softer platforms, whereas the Stiff Col-Tgel had the lowest ROS expression irrespectively of the concentration of 5-Aza-CR.Yet, ADSCs treated with 5-Aza-CR exhibited an upregulation of ROS expression, especially in the Med and Stiff Col-Tgels (*P* < 0.001). These results suggest that cells sensitized with the epigenetic modulator were more readily activated in a mechanical restricted microenvironment.

### Cell remodeling

**Figure 4:**
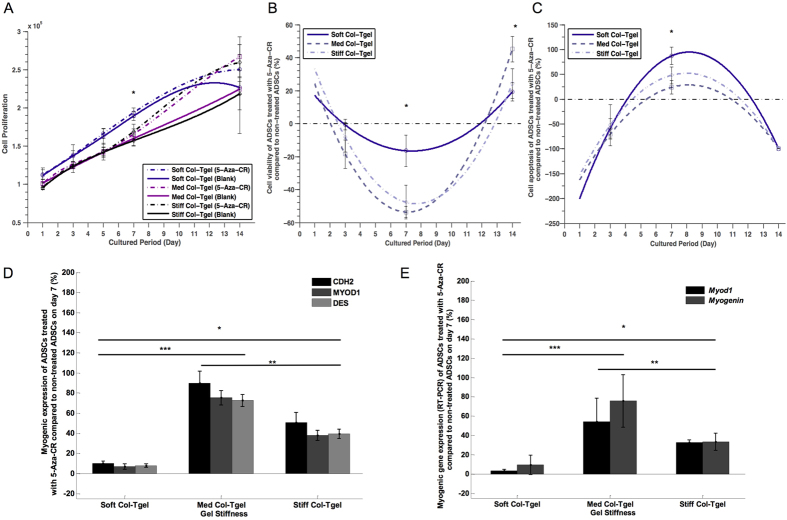
Cell remodeling. ADSCs were encapsulated in the Soft, Med, and Stiff Col-Tgels and grown with or without 5-Aza-CR. (**A**) Cell counting. (**B**) CCK-kit8 assay. (**C**) Caspase 3/7 staining. Cell numbers remained stable after the addition of 5-Aza-CR and a significant number of new cells were generated by day 14. Although significant apoptosis was observed on day 7, it was transient, and 5-Aza-CR-treated group exhibited a relatively lower degree of apoptosis than untreated group. Regardless of the presence of 5-Aza-CR, an increase in gel stiffness boosted cell apoptosis and reduced cell doubling rate. (**D**) Immunostaining (CDH2, MYOD1, and DES proteins). (**E**) RT-PCR analysis (*Myod1* and *Myog* genes) of myogenic precursor markers. Highest levels of myogenic marker expression during the conversion of ADSCs to myoblast-like cells were observed in the Med Col-Tgel, while lower expression was detected in the Stiff and Soft Col-Tgels. Incubation with the epigenetic modulator 5-Aza-CR potently enhanced the expression of myogenic markers but did not alter the tendency of maximum myogenic differentiation to occur in the Med Col-Tgel, *i.e.*, in the microenvironment of approximately 15 kPa that corresponds to the elasticity of native muscle extracellular matrix. Values (excluded cell counting) in the treated group were normalized by corresponding values in the non-treated group and scaled between 100% (upregulation) and −100% (downregulation). Data are presented as the mean ± standard deviation of 4 samples and statistical differences (Tukey-Kramer HSD test) are indicated as follows: *P < 0.05, **P < 0.01, and ***P < 0.001.

Cell proliferation and cell death are important events regulating tissue regeneration and development (38). Therefore, cell counting (Fig. 4A), CCK-kit8 (Fig. 4B), caspase 3/7 staining (Fig. 4C), and live/dead cell staining (Fig. S5) were performed to identify the number of viable cells. As illustrated in Fig. 4A, the number of cells with or without treatment of 5-Aza-CR was stably increasing over the culturing period in all gel types. This could be attributed to a competition between the doubling rate (Fig. 4B and S5) and the apoptotic rate (Fig. 4C and S5), as well as to the impact of matrix rigidity. Regardless of the treatment with 5-Aza-CR, the increase in gel stiffness was associated with the reduction in the cell doubling rate and cell viability as well as with decreased levels of cell death (Fig. 4B,C and S5). Although apparent cell death was observed on day 7 in all gel conditions (Fig. 4B and S5), possibly due to p53-dependent apoptosis (39), the surviving cells proliferated actively and generated new cell populations, as evidenced by a stable total cell number and a comparable number of live cells on day 7. As expected, these numbers subsequently increased by day 14 in culture (Fig. 4A and S5). Intriguingly, we noted that less cell apoptosis was observed in those treated with 5-Aza-CR on day 14. Overall, these results suggest that 5-Aza-CR had a significant positive effect on cell proliferation, with the doubling rate of surviving cells being able to compensate fully for the cell loss due to apoptosis.

Apart from its effect on intracellular processes, the cellular microenvironment may also influence cell-cell interactions^28^,^37^ and dictate the cell destiny^38^. Thus, additional effects of 5-Aza-CR on myogenic differentiation in ADCSs grown on different matrices were assessed by immunostaining and RT-PCR (Fig. 4D,E and S6). Immunostaining for CDH2, MYOD1, and DES revealed that 5-Aza-CR enhanced the expression of these myogenic markers regardless of the type of gel (Fig. 4D). Furthermore, consistent with a key role of the matrix in the cell commitment toward the myogenic lineage, the largest augmentation of myogenesis was found in cells cultured in the Med Col-Tgels in both 5-Aza-CR-treated and untreated cells. ADSCs cultured in the Soft Col-Tgel demonstrated an insignificant amount of myoblast maturation as compared to the Med and Stiff Col-Tgels. Our hypothesis that the rigidity of matrix plays a crucial role in regulating cell commitment to a particular lineage was further strengthened by *Myod1* and *Myog* gene expression data (Fig. 4E). As in the above experiments, 5-Aza-CR increased the expression of these myogenic genes, especially in ADSCs cultured in the Med Col-Tgels. It is worth noting that the myogenic marker was expressed throughout the duration of the whole experiment with abundant expression was observed in the Med Col-Tgel (Fig. S6D). Therefore, the Med Col-Tgel was chosen as the carrier for cell-based delivery to restore critical muscle defects.

### Functionalized cell-based tissue engineering and regeneration

**Figure 5:**
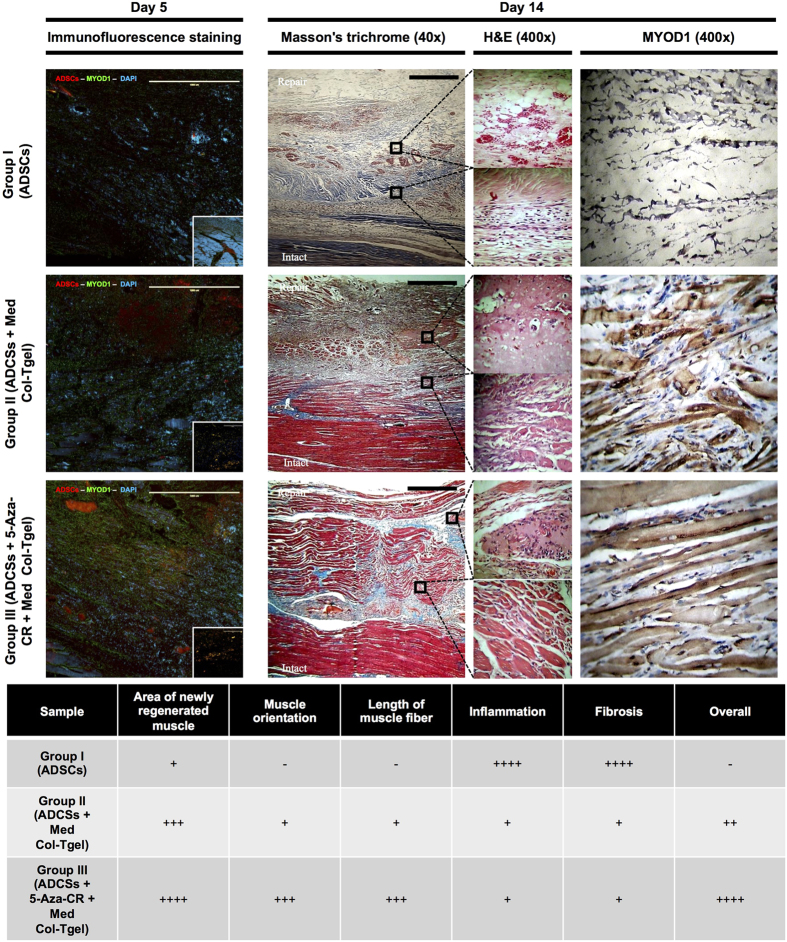
*In vivo* cell-based tissue engineering. Histological evaluation of the following groups of ADSCs 5 and 14 days after their introduction to the injury site: ADSCs only (group I), ADSCs in the Med Col-Tgel (group II), ADSCs in the Med Col-Tgel supplemented with 5-Aza-CR (group III). H&E, Masson’s Trichrome, and MYOD1 immunostaining were performed. Muscle formation was evaluated by assessing the new muscle formation area, and myofiber orientation and length, while inflammation and fibrosis were considered to have a negative effect on the muscle formation. As demonstrated by MitoTracker® labeling, group I ADSCs were untraceable at the injury site, while ADSCs remained visible. Immunofluorescence staining showed that ADSCs delivered by Col-Tgel containing 5-Aza-CR trans-differentiated into myoblast-like cells. On day 14, treatments with group II and III ADSCs led to the emergence of large areas of new muscle formation, while group I ADSCs were not associated with appreciable muscle regeneration. Group III ADSCs promoted the appearance of largest areas of new muscle formation and maturation, with minimal contamination by fibroblasts (Scale bars, 1000 μm).

To study the effectiveness of combined treatment with ADSCs and the epigenetic modulator 5-Aza-CR that encapsulated in Col-Tgel for muscle tissue regeneration, animals were euthanized on days 5 and 14 after the delivery of the prepared sample to the TA muscle injury site. Hematoxylin and eosin (H&E), Masson’s Trichrome, and MYOD1 immunohistochemistry/immunofluorescence staining procedures were performed, and a semi-quantitative histological score of the new muscle tissue formation was obtained taking into account such parameters as area, length, orientation, and maturation of newly formed myofibers (Fig. 5). MitoTracker® was used to localize the delivered ADSCs as well as to track whether ADSCs trans-differentiated into myoblast-like cells. The immunofluorescence staining revealed that ADSCs delivered without the carrier (*i.e.*, Group I in Fig. 5) were absent from the recovering injury site, whereas the ADSCs applied with Col-Tgel (*i.e.*, Group II and III in Fig. 5) were well represented there. The insert on the panel with the immunofluorescence staining of Group I demonstrated that ADSCs delivered without the carrier invaded the intact muscle, indicating the importance of scaffold in localization of the delivered cells. Furthermore, data from ADSCs labeled by MitoTracker® and counterstained for MYOD1 suggested that myogenesis was minimal in Group I, while the maximum myogenic differentiation was observed in Group III. Inserts on the panels with the immunofluorescence staining of Group II and III also show that ADSCs treated with 5-Aza-CR (Group III) were trans-differentiated into muscle cells more significantly than untreated cells (Group II).

On day 14, we detected a significant infiltration of fibroblasts, adipocytes, inflammatory cells and revealed neovessel invasion, necrosis, hemorrhage, and a minimal extent of the new muscle tissue formation in group I (i.e., ADSCs only, Fig. 5) and in the vehicle group (Group S1, Fig. S7). Although the Med Col-Tgel only condition (Group S2, Fig. S7) prevented infiltration of fibroblasts, no significant muscle tissue formation was detected in this group. On the contrary, substantial new muscle tissue formation was revealed in Group II and Group III, indicating the importance of the scaffold in supporting cell invasion. However, introduction of ADSCs with Col-Tgel scaffold and 5-Aza-CR promoted potentiated muscle tissue formation to a greater extent compared to the condition with Col-Tgel but without 5-Aza-CR. H&E images at 400 × magnification showed that cells were able to survive within the gel. The use of Col-Tgel in combination with 5-Aza-CR enhanced cell survival, upregulated the invasive capability of cells, and promoted cell differentiation within the gel. Furthermore, the newly formed muscle fibers in the group treated with ADSCs and a combination of 5-Aza-CR and Col-Tgel were found to be well aligned and parallel to intact muscle fibers at the repair site, while a fairly random organization of the injury site was observed in the group that was not treated with 5-Aza-CR. We observed that newly formed muscle fibers were about 180 ± 20 μm in length and contained myonuclei along myofibers in the 5-Aza-CR-treated group. At the same time, myofibers in the untreated group were shorter (about 60 ± 40 μm) and had centrally located multinucleated myonuclei. Fibrosis was also minimally observed in Group III. In addition, blood vessels were observed to distribute evenly throughout the new muscle area, especially in the group that was incubated with 5-Aza-CR. The results obtained with the animal TA muscle injury model suggest that an optimal functional matrix (*i.e.*, ADSCs in Col-Tgel with 5-Aza-CR) is necessary for rapid muscular architecture restoration.

## Discussion

Recent rapid developments in the field of regenerative medicine and functional tissue engineering have highlighted the importance of stem cells. Many commercial and academic laboratories have contributed significant resources to the research on embryonic and adult tissue-derived stem cells with the aim of developing methods of rapid tissue repair after transplantation. However, after two decades, these treatments are still at the experimental stage because of the questionable efficacy of such cells in the adult body. 5-Aza-CR, an epigenetic modulator, has been utilized previously to improve cell reprogramming through the activation of gene expression^39^,^40^, but the role of the microenvironment in reprogramming has been generally neglected. Recent studies revealed a prominent role of the microenvironment in the regulation of progression of specific tissue lineages toward the terminal phenotype. A better understanding of how the microenvironment cues influence cell fate would allow to improve the ability of the artificially engineered environment to control cell fate and functional tissue regeneration^41^,^42^. Therefore, in this study, we evaluated the interactions between the epigenetic modulator 5-Aza-CR and the 3-D microenvironment on cellular responses by utilizing tunable Col-Tgels. We also examined the stimulating role of 5-Aza-CR on the trans-differentiation of precursor cells into myoblasts-like cells in Col-Tgels to provide a more comprehensive picture of the influence of matrix components on cell reprogramming and muscle development. Furthermore, we demonstrated that an adjustment of one or more material parameters predictably altered cellular responses. Most importantly, we observed that severe muscle injuries could be rapidly restored to its normal muscular architecture in our *in vivo* TA muscle injury model following the application of a suitable matrix (Med Col-Tgel) and effective doses of 5-Aza-CR (1.25–12.5 ng).

There are several concerns regarding the clinical applications of epigenetic drugs such as DNA methylation inhibitors. 5-Aza-CR is known to be a cytotoxic agent that induces cell cycle arrest and apoptosis by upregulating p21 and/or p53^43^,^44^. However, in this study, we showed that incubation with 5-Aza-CR did not induce any permanent cell cycle arrest in cells cultured for all gel types (Fig. 4 and S5). This conclusion stemmed from our finding that the number and viability of ADCSs were higher after treatment with 5-Aza-CR compared to data from untreated groups on day 14 (Fig. 4 and S5). Although 5-Aza-CR stimulated transient apoptosis on day 7, the surviving cells became more resilient and had an augmented capacity for self-renewal in the presence of the drug (Fig. 4 and S5). Furthermore, the cells treated with 5-Aza-CR exhibited considerably reduced apoptosis on day 14 (Fig. 4 and S5), indicating that putative cytotoxicity induced by 5-Aza-CR has no persistent adverse effects on cell survival. Previously, Pennarossa *et al.*^14^ have demonstrated that alteration of the differentiation state induced by 5-Aza-CR is transient and does not involve cellular toxicity. In our *in vivo* studies, we did not detect appreciable toxic effects of 5-Aza-CR on treated animals (Fig. 5). Nevertheless, we would like to note that the identified optimal dose range (i.e. from 1.25 ng to 12.5 ng) that promoted cell reprogramming (Figs 1 and 2) and myogenesis (Figs 5 and 6) is extremely low compared to doses currently used in pre-clinical (>25 ng) and clinical (>10.7 mg) settings for cell reprogramming and cancer treatment, respectively^14^,^45^. Therefore, one single combined treatment used in this study is not only cost effective but also appears to be safe.

2-D *in vitro* studies^14^,^46^ have shown that 5-Aza-CR can rapidly reactivate the expression of genes that have undergone epigenetic silencing and facilitate cell reprogramming and development. Yet, the results of 2-D *in vitro* studies should be re-examined in more physiologically relevant 3-D microenvironments, as cells cultured on 2-D surfaces might acquire altered functional characteristics^28^,^47^. Therefore, we adopted a more physiologically relevant 3-D microenvironment to study the effects of 5-Aza-CR on cell reprogramming and development. Similar to 2-D *in vitro* results, we found that 5-Aza-CR reinforced the flexibility of cells, allowed direct reprogramming of ADSCs into myoblast-like cells, as demonstrated by both *in vitro* and *in vivo* experiments conducted in this study (Figs 4D,E and 5 and S6). However, there were some discrepancies between 2-D and 3-D *in vitro* studies. For example, ADSCs originally formed a monolayer and displayed a standard elongated morphology in the 2-D setting, while ADSCs embedded in the 3-D environment were found to be less elongated than cells on the 2-D surface (Fig. 1 and S7). Furthermore, Pennarossa *et al.*^14^ reported that cell proliferation rapidly decreased after the exposure to 5-Aza-CR as compared to the non-treated group. In contrast, our results indicated that cell proliferation of 5-Aza-CR group remained similar to the non-treated group (Fig. 4A–C and S5). Although there was a transient high apoptosis after the exposure to 5-Aza-CR, the cell numbers did not change significantly as compared to non-treated group. This might be due to the competition between the doubling rate (Fig. 4A,B and S5) and the apoptotic rate (Fig. 4C and S5), as well as to the impact of matrix rigidity. These discrepancies could arise from the fact that Pennarossa *et al.*^14^ adopted a 2-D microenvironment in their study, whereas we used a substrate that better mimicked the physiological microenvironment in terms of 3-D spaces and matrix rigidity. This circumstance could cause differences in the cell doubling rate between the two studies. Thus, we demonstrated that the microenvironment plays an important role in shaping cell characteristics.

The degrees of compliance between different Col-Tgels are determined by, but not limited to, the binding sites, pore sizes, porosity, nutrient diffusion rate, pH, oxygen tension, and interstitial pressure. Studies^18^,^48^,^49^ have repeatedly showed that matrix rigidity and ligand density are highly coupled variables that dictate mean cell responses ranging from cell spreading to cell shape and molecular organization. Engler *et al.*^48^ have also demonstrated that cells spreading on soft gels are relatively unresponsive to ligand density, but the stimulation on stiffer substrates is far greater in comparison. Hence, the intercellular microenvironment cues are interrelated. Furthermore, the endogenous extracellular matrix remodeling might occur as cells dynamically degrade, assemble and deposit their own matrix^18^. Therefore, in this study, we divided the matrix compliance into three different level of stiffness to study the influence of different microenvironment. Understanding fundamental mechanisms of how cells interact with the microenvironment could eventually result in predictive control of cell fate and function, which will enable rational design strategies for tissue engineering and identify avenues for cell-based regenerative therapies. In addition, we introduced an easy-to-built high throughput yet physiologically relevant system for studying the response of cells to the multiple environmental parameters.

A number of outstanding reports^18^,^50^,^51^ have provided new insights into the important role of microenvironmentsignals transmitted from the ECM to cells, in modulation of cellular self-renewal and differentiation. Our data extend these findings by indicating a role of the microenvironment in epigenetic reprogramming that consequently influences cellular responses. We found that 5-Aza-CR activated quiescent cells and strengthened the capacity of cells to self-renew in the proper microenvironment (Figs 1 and 2, and S3). Nonetheless, cells activated by 5-Aza-CR were more sensitive to changes in the microenvironment (Fig. 3), and exhibited a significant increase in ROS expression, particularly if they were grown in stiffer matrices (Fig. 3 and S4). The stimulated intracellular signaling further influence and regulate the downstream cell reprogramming. Intriguingly, the aforementioned events were less vividly observed in the similar gel condition if without the activation of 5-Aza-CR. Likewise, the addition of epigenetic drug into our models did not alter the finding that spontaneous myogenic differentiation occurred primarily in the Med Col-Tgel (Fig. 4D,E and S6). The resistance sensed by a cell when it deforms the ECM is described by the elastic constant of the matrix or microenvironment^18^,^28^. The elasticity of the Med Col-Tgel used in this study is about 10–20 kPa, which mimics striated muscle elasticity (∼8–17 kPa)^50^ and leads to the emergence of spindle-shaped cells similar to myoblasts (Fig. 3). The capacity to undergo the trans-differentiation was further substantiated in our *in vivo* study, where labelled ADSCs were able to express a myogenic marker (Figs 4D,E and 5 and S6). In contrast, ADSCs trans-differentiated into myoblast-like cells were least detectable in the microenvironment that did not favor myogenic differentiation, even in the presence of 5-Aza-CR (Figs 4D,E and 5 and S6). Thus, both chemical modulators and microenvironment cues are equally important for cell reprogramming and development.

Better engineered ECMs that can control cell behavior through physical as well as molecular interactions may further extend the capabilities of tissue engineering^52^. In the present study, we showed that ADSCs trans-differentiated into myoblast-like cells could be finely tuned provided the cells were grown in the optimal microenvironment (*i.e.*, the Med Col-Tgel as shown in Fig. 4D,E and S6). It is worth noting that although the ADSCs and animal model used in this study initially lacked the self-regeneration ability because of age. Yet, we noted that this epigenetic modulator significantly potentiated myogenesis in the Med Col-Tgel, as demonstrated in both *in vitro* and *in vivo* models (Figs 4D,E and 5 and S6). The Col-Tgel used in this study not only regulated cell destiny, but also localized the transplanted stem cell niche and controlled the release rate of chemical stimuli (*i.e.*, 5-Aza-CR) to amplify the regenerative capacity (Fig. 5). While the meta-analysis revealed that 1 × 10^8^−10^9^ of cells were needed for cell transplantation to obtain clinically significant cell numbers^53^, we demonstrated that 1 × 10^5^ of localized cells were sufficient to restore severe muscle injuries in this study (Fig. 5). Moreover, Col-Tgel possesses flexible handling properties that allow it to conform to any irregularly shaped defect. Such scaffold provides a temporary blueprint for subsequent regeneration, while appropriate material chemistry and substrate architecture support cell reprogramming, survival, self-renewal, and cell invasion as well as prevent fibrotic scar tissue formation. ADSCs treated with 5-Aza-CR reduced the progression of fibrosis through accelerating muscle tissue formation, as depicted in Fig. 5. Precise control over the scaffold shape, transplanted cells at the repair site, release of epigenetic stimuli, and cell destiny is critical to ensure proper cellular remodeling and accelerate tissue repair to its normal architecture.

The advancement on cell plasticity is inevitable for accelerating cell regeneration at the defect site. Nonetheless, it has become evident that adjuvant materials for restoring genetic or accidental muscle defects to its normal architecture need to mimic the physiological environment to support and control the release of chemical stimuli to amplify the regenerative capacity. The inexpensive and scalable Col-Tgel system highlights a novel approach in regenerative medicine, as it can be integrated either as a testing model or as a potential carrier for cells and chemical stimuli. Easy accessibility of fat tissue has made ADSCs an attractive cell source choice for tissue engineering and regenerative medicine. It is clear that microenvironment factors can significantly influence the overall behavior of ADSCs populations, although the details of many regulatory mechanisms have not yet been elucidated. Cell delivery by a protective gel enables cells to localize at the repair site. Furthermore, loading a diffusible epigenetic drug into the carrier can also promote the stimulation of cell self-renewal and flexibility of the transplanted cells. The spatial and temporal control of these features would help enhance tissue regeneration. Therefore, this study may serve as the basis for future attempts to stimulate repair and regeneration of other tissues by similar approaches that would collectively advance the contemporary toolkit of tissue engineering and regenerative medicine.

## Materials and Methods

### Materials and Reagents

Unless otherwise stated, all chemicals and reagents were purchased from Sigma-Aldrich (St. Louis, MO). Full details on the preparation of microbial transglutaminase (TG) and gelatin gel (Gel) can be found elsewhere^18^,^54^,^55^.In this study, the final elasticity of gels were determined through conventional unconfined compression test^18^, where they were kept to be 0.9 ± 0.1 kPa (Soft), 15 ± 5 kPa (Med), and 40 ± 10 kPa (Stiff).

### Isolation of ADSCs

ADSCs were isolated from Fisher 344 retired breeders (18–20 months old) as described^56^. Briefly, adipose tissues were dissected from the abdomen and digested with bacterial collagenase type II (Worthington, Lakewood, NJ). The digested cells were filtered and washed twice with the medium consisting of high glucose Dulbecco’s modified Eagle medium (DMEM, Mediatech, VA) supplemented with 2% (v/v) penicillin-streptomycin (PS, Mediatech, VA). Then, two millions of cells were plated on individual 60 mm Petri dishes containing DMEM growth medium supplemented with 10% (v/v) fetal bovine serum II (FBS, Thermo Scientific, MD) and 1% (v/v) PS in a humidified atmosphere of 95% O_2_/5% CO_2_ at 37 °C. The medium was changed every 2–3 days. When cells reached 70% confluence, they were sub-cultured using 0.25% trypsin and 0.02% ethylenediaminetetraacetic acid (EDTA) solution.

### *In vitro* 3-dimensional Col-Tgel model

*In vitro* 3-D Col-Tgel cell construct was created to evaluate the influence of the ECM on cellular functions, as described^18^. Briefly, 100 μL of the gel solution was evenly mixed with 2 × 10^5^ ADSCs between 6^th^ to 10^th^ and 5 μL of transglutaminase (TG) to create Col-Tgel cell mixtures. Twenty μL of the Col-Tgel cell mixture was seeded into each well of 48-well suspension plate and growth medium was added after the gel solidified through chemical cross-linking. The growth medium was supplemented with various concentrations of 5-Aza-CR to study the effects of this epigenetic modulator on cell reprogramming in the Soft, Med, and Stiff Col-Tgels. Samples were collected for analysis on days 1, 3, 5, 7, and 14.

### Oil red O staining and colorimetric assay

ADSCs were visualized and quantified using the Oil red O assay, as described^24^,^57^. Briefly, Col-Tgel-Cell construct was fixed with 10% neutral formalin, washed twice with 60% isopropanol and underwent a complete immersion in a working solution of Oil red O (0.3% Oil red O in isopropanol) for 2 hours. Prior to photography, stained samples were washed twice with dH2O to remove nonspecific binding. Positive red-orange staining represents lipid deposition by encapsulated ADSCs. Images were captured to analyze the unique characteristics of ADSCs. After photography, dH_2_O was removed, and samples were incubated in 500 μL of isopropyl alcohol for 2 hours. The supernatant was collected and absorbance was measured by a multiplate reader (Molecular Devices, Sunnyvale, CA).

### Histochemical staining

Aging cells were assessed using a β-Gal staining kit (Invitrogen, Carlsbad, CA) strictly according to the manufacturer’s instructions. Briefly, Col-Tgel-Cell constructs were fixed and incubated in the β-Gal reaction buffer for 2 hours at 37 °C. Positive staining was identified using light microscopy (Nikon, Japan). Immunostaining was performed at room temperature to characterize cell plasticity, cell proliferation, cell-matrix and cell-cell interactions, and myogenic differentiation, as described previously^18^. Briefly, the Col-Tgel-Cell construct was fixed in 10% neutral formalin solution for 10 min, incubated in peroxidase suppressor solution (Thermo Scientific, Newington, NH) for 30 min, and then in the blocking buffer (5% BSA in the mixture of Tris-buffered saline and Tween-20) for 30 min. Next, the sample was incubated overnight in the primary antibody working solution as listed in Table S1, followed by incubation in the biotinylated secondary antibody working solution (1:800 in blocking buffer; Sigma, St. Louis, MO) for 1 h. The Col-Tgel-Cell construct was incubated in the SPPU working solution (ultrasensitive streptavidin–peroxidase polymer in blocking buffer 1:800; Sigma, St. Louis, MO) for 30 min. The color reaction was revealed by incubating the sample in the 3,3′-diaminobenzidine working solution (metal-enhanced substrate solution diluted with the stable peroxide buffer, 1:10, Thermo Scientific, Newington, NH) and the reaction was stopped by washing the sample with TBST when the desired color was developed (~5 min). Microscopic analysis was performed to examine positive staining.

### Fluorescence staining

Cell cytoskeleton organization, and cell responses to stress and cell viability were analyzed using an F-actin staining kit (Invitrogen, Carlsbad, CA), a reactive oxygen species (ROS) staining kit (Life Technologies, Gaithersburg, MD), a LIVE/DEAD Viability/ Cytotoxicity Kit (Life Technologies, Gaithersburg, MD), and CellEvent™ Caspase-3/7 Green ReadyProbes® Reagent (Life Technologies, Gaithersburg, MD), respectively. The assays were performed strictly according to the manufacturers’ recommendations. Briefly, the Col-Tgel-Cell construct was rinsed with Tris-buffered saline with 0.05% Tween-20 (TBST, pH 7.4), fixed in 10% neutral formalin solution (VWR International, Radnor, PA) for 10 min, and incubated in the blocking buffer (5% BSA in TBST) for 30 min. Then, the samples were incubated with the prepared working solution according to the recommended concentration. After a 2-h incubation at 4°C, the Col-Tgel-Cell construct was counterstained with DAPI (Biotium, Hayward, CA) for 5 min and visualized under an EVOS fluorescence microscope (Advanced Microscopy Group, Bothell, WA).

### Proliferation and Cytotoxicity Assays

Cell counting and cell viability were performed to determine the cell growth and viability of ADSCs encapsulated in Col-Tgels by using a cell counter and a Cell Counting Kit-8 (CCK-8, Dojindo Molecular Technologies, Rockville, MD), as described^18^. Briefly, cells were released from Col-Tgel-Cell constructs using 20 units of bacterial collagenase type II (*Worthington, Lakewood, NJ*) prior to counting by a cell counting machine (Beckman Coulter, Brea, CA). For the CCK-8 assay, 500 μL of the CCK working solution (CCK-8 stock solution in growth medium, 1:40) was directly applied to the Col-Tgel-Cell construct for 4 h. Subsequently, 100 μL of the supernatant was transferred into 96-well plates and the absorbance was measured in a multiplate reader at 450 nm (Molecular Devices, Sunnyvale, CA).

### Total RNA extraction and RT-PCR analysis of the gene expression

Total ADSCs RNA grown in Col-Tgels was extracted using Trizol reagent (Invitrogen, Carlsbad, CA) according to the single-step acid–phenol guanidinium extraction method described previously^18^,^58^, and real-time PCR was performed with one-step SYBR green reagents according to the manufacturer’s protocol (Bio-Rad, Hercules, CA). In brief, Col-Tgel-Cell constructs were cultured in the media with or without 5-Aza-CR in 48-well suspension plate for 1, 3, 7, and 14 days. The sample was collected in 1.5-mL tubes containing 1 mL of Trizol reagent. After a thorough homogenization and removal of insoluble substances, the sample was treated with 1-bromo-3-chloropropane. Total RNA was precipitated using chilled ethanol and the acquired RNA sample was treated with RNase-free water. RNA concentration was quantified spectrometrically at 260 and 280 nm, and the purity was determined at 230 and 260 nm (Implen GmbH, Munich, Germany). The expression of target genes was normalized according to the *GAPDH* gene reference by using the ΔΔCt method. The primers (ValueGene Inc, San Diego, CA) used for amplification are listed in Table S2.

### *In vivo* cell-based delivery

To study the process of muscle regeneration in a controlled and reproducible manner, a 6-mm diameter myectomy lesion in the TA muscle of each rat was created using a biopsy punch. All animals used in this study were treated with great care and in accordance with the protocols approved by the *Institutional Animal Care and Use Committees* (IACUC) of the University of Southern California. Thirty 18-20-month-old Fischer-344 rats (NCI, Rockville, MD) were randomly divided into 3 groups: PBS only (Group S1), Med Col-Tgel only (Group S2), ADSCs only (Group I), ADSCs with Med Col-Tgel (Group II), and ADSCs with 5-Aza-CR and Med Col-Tgel (Group III). The preparation of Col-Tgel with 5-Aza-CR was made by mixing 100 μL of pre-heated gel solution and 5 μL of TG with 12.5 ng of 5-Aza-CR. Each animal was infused with 100 μl of the prepared sample directly into the induced TA muscle injury site, using a G-27 needle. Six rats of each group were euthanized after 5 or 14 days following the injection. Muscle tissue was dissected, fixed, decalcified, and sectioned for H&E staining, Masson’s Trichrome Staining, and immunohistochemistry.

### Image analysis

Four randomly selected fields per sample were analyzed. A code programmed in Matlab (MathWorks Inc., Natick, MA) was used to calculate the percentages of positively stained cells by calculating the average area of dark brown/green/red spots as a fraction of the total area of the sample from four randomly selected fields in duplicate samples.

### Statistical Analysis

Statistical differences were determined using ANOVA followed by the Tukey-Kramer honest significant difference (HSD) test for pairwise comparisons (SAS, Cary, NC). Values of *P* < 0.05 were considered to indicate statistically significant differences as follows: *P < 0.05, **P < 0.01, and ***P < 0.001. Data obtained from cells encapsulated in the Col-Tgel containing 5-Aza-CR were normalized with respect to those in the medium without the drug and scaled to vary from −100% to + 100% to demonstrate the positive and negative effects of the treatment.

## Significance Statement

In this study, we described the modulation of cellular functions in the 3-D microenvironment by an epigenetic modulator, examined scaffold effects on cellular responses, and explored how the material-cell-based therapy can be tuned to optimize tissue regeneration. Our results suggest that cellular plasticity can be enhanced by epigenetic reprogramming and cell destiny can be controlled by the microenvironment. Cell-based therapy employed in this study amplified the regenerative capacity of severely injured muscles and induced self-regenerative functions that are usually gradually lost because of aging. In summary, our study provides new insights into the regulation of self-renewal and differentiation of pluripotent cells, and encourages the translation of cell-based therapies into clinical applications.

## Additional Information

**How to cite this article**: Tan, S.J. *et al.* Muscle tissue engineering and regeneration through epigenetic reprogramming and scaffold manipulation. *Sci. Rep.* **5**, 16333; doi: 10.1038/srep16333 (2015).

## Supplementary Material

Supporting Information
