# Supplementary material for: Muscle tissue engineering and regeneration through epigenetic reprogramming and scaffold manipulation
Source: Sci Rep. 2015 Nov 9;5:16333. doi: 10.1038/srep16333 (PMC4637833; doi:10.1038/srep16333)
Supplement: Supporting Information [file srep16333-s1.pdf]

Muscle Tissue Engineering and Regeneration through Epigenetic Reprogramming and Scaffold Manipulation

S.J. Tan[a], J.Y. Fang[b], Y. Wu[b], Z. Yang[b], G. Liang[c], B. Han[a,b,*]

[a] Department of Biomedical Engineering, Viterbi School of Engineering, University of Southern California, Los Angeles, CA

[b] Division of Plastic and Reconstructive Surgery, Department of Surgery, Keck Medical School, University of Southern California, Los Angeles, CA

[c] Department of Urology, Keck School of Medicine, University of Southern California, Los Angeles, CA 90089, CA

[*] Correspondence should be addressed to

Han, Bo (bohan@usc.edu)

University of Southern California

1333 San Pablo St., BMT 302A

Los Angeles, CA 90089-9112

USA

Tel: +1 (323) 442-2242

Fax: +1(323) 442-5956

Keywords

ECM (extracellular matrix), 5-Azacytidine, Myogenesis, Cell encapsulation, Demethylation, Cell-based therapy, Epigenetic reprogramming

Supporting Information

SI figure

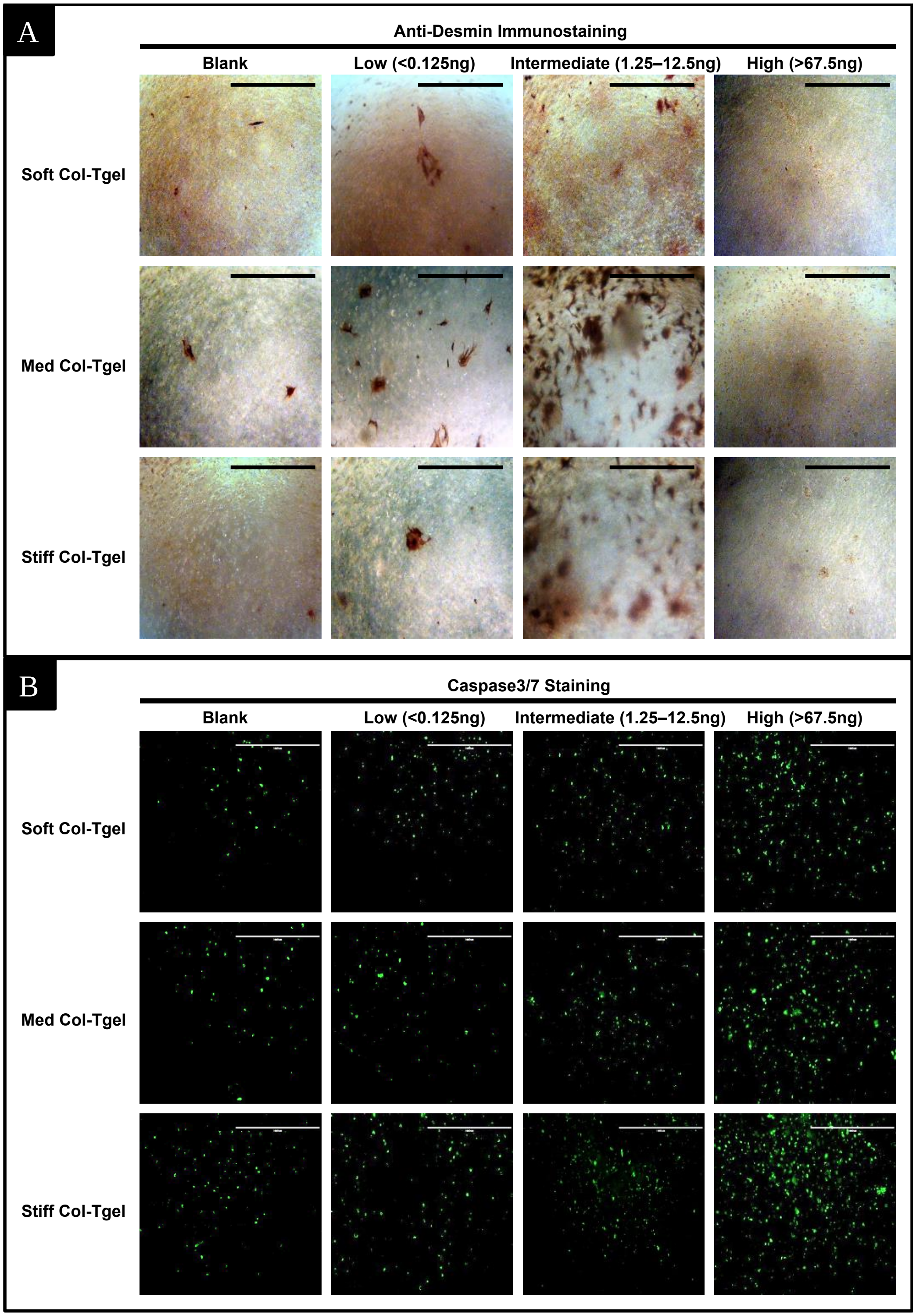

Fig. S1. **Effective dosage of 5-Aza-CR on cell reprogramming.** (*A*) DES immunostaining. (*B*) Caspase 3/7 staining. Adipose derived stromal cells (ADSCs) treated with or without low (<0.125ng), intermediate (1.25 – 12.5ng) or high (>67.5ng) dose of 5-Aza-CR were encapsulated in Soft (0.9 ± 0.1 kPa), Med (15 ± 5 kPa) and

Stiff (40 ± 10 kPa). The low dose of 5-Aza-CR used in this study did not enhance significant trans-differentiation of ADSCs into myoblasts-like cells. ADSCs treated with intermediate dosage of 5-Aza-CR were dramatically trans-differentiated into myoblasts-like cells, especially those in the Med Col-Tgel. High dose of 5-Aza-CR did not promote trans-differentiation, but programmed ADSCs to death regardless of gel stiffness (Scale bars, 1000 μm).

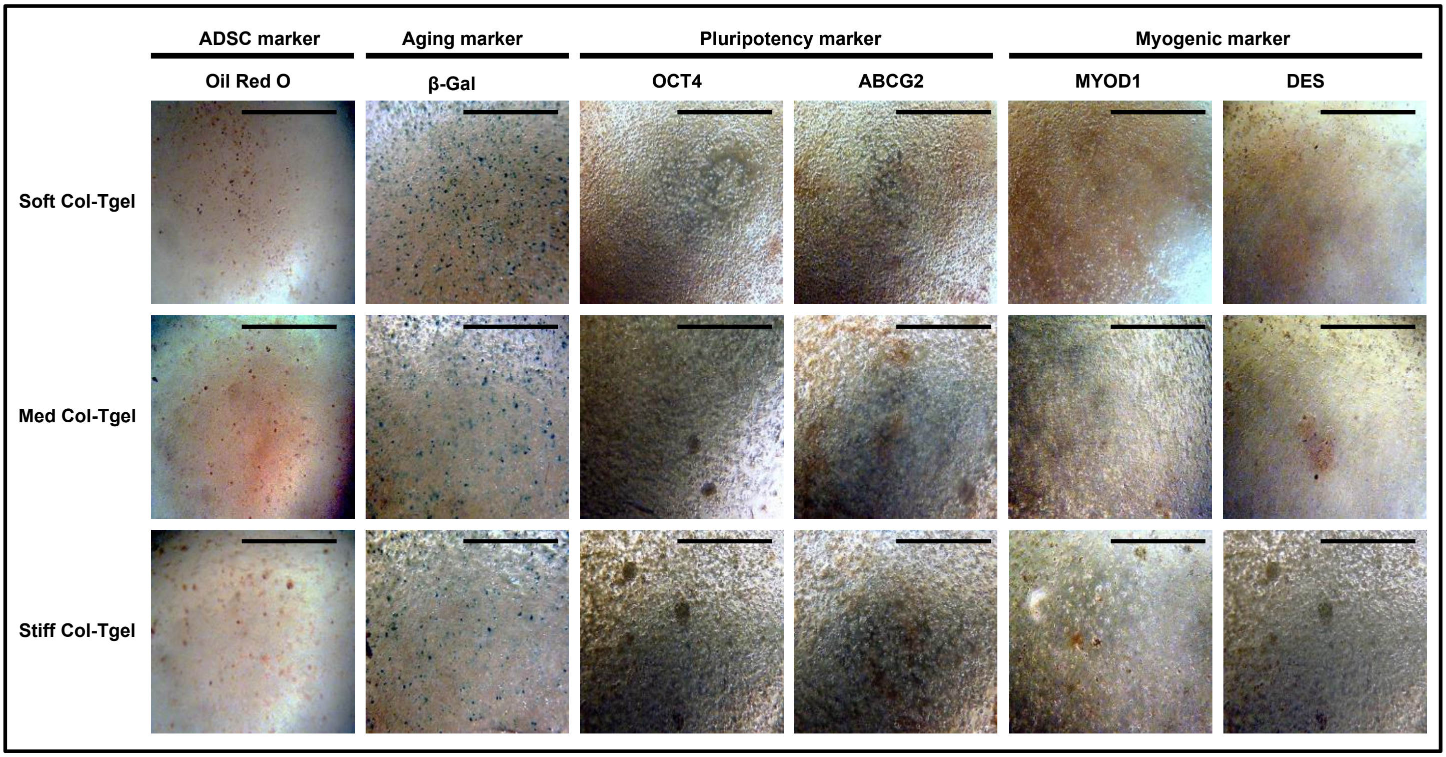

Fig. S2. **The original phenotypes of ADSCs.** (*Left to right*) Oil red O and β-Gal staining, and OCT4, ABCG2, MYOD1 and DES immunostaining. ADSCs were encapsulated in the Soft, Med, or Stiff Col-Tgel and grown without 5-Aza-CR. ADSCs were originally not stem cells and myoblast-like cells (Scale bars, 1000 μm).

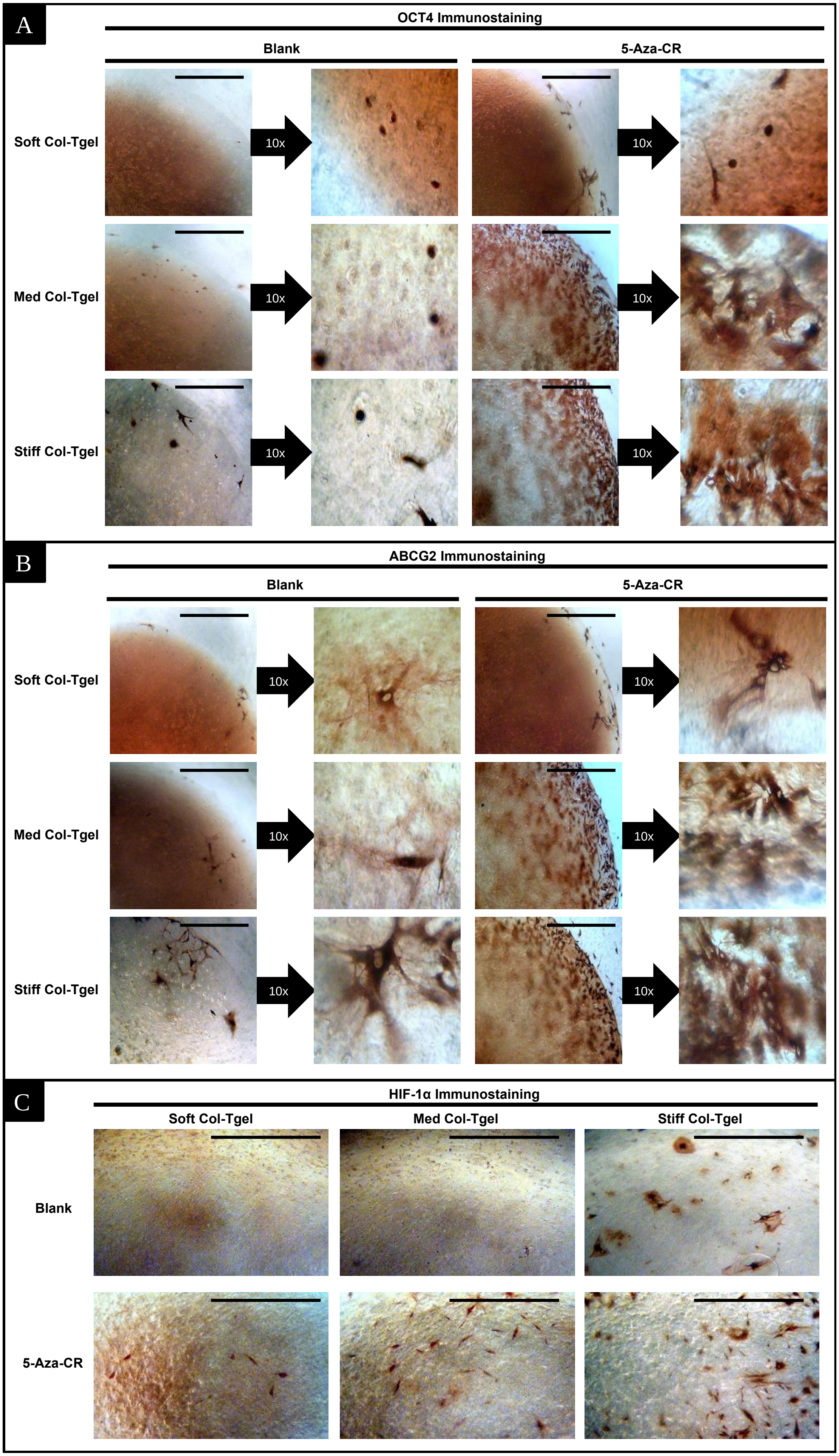
A
OCT4 Immunostaining
Blank
5-Aza-CR
Soft Col-Tgel
Med Col-Tgel
Stiff Col-Tgel
10x
B
ABCG2 Immunostaining
Blank
5-Aza-CR
Soft Col-Tgel
Med Col-Tgel
Stiff Col-Tgel
10x
C
HIF-1α Immunostaining
Soft Col-Tgel
Med Col-Tgel
Stiff Col-Tgel
Blank
5-Aza-CR

Fig. S3. **Activation of silent gene(s).** ADSCs were encapsulated in the Soft, Med, or Stiff Col-Tgel and grown with or without 5-Aza-CR. (*A*), (*B*) and (*C*) Immunostaining of OCT4, ABCG2 and HIF-1α, respectively. The supplement of 5-Aza-CR showed up-regulation of the expression of OCT4 and ABCG2 of ADSCs, where the highest was found in stiffer gels (*i.e.*, Med and Stiff Col-Tgels) and the lowest was observed in the Soft Col-Tgel. Besides the activation of stem cells markers, the HIF-1α immunostaining revealed that HIF-1α was stimulated by the addition of 5-Aza-CR compared to those non-treated cells. The HIF-1α expression was found to be enhanced by gel stiffness, regardless of the presence of 5-Aza-CR (Scale bars, 1000 μm).

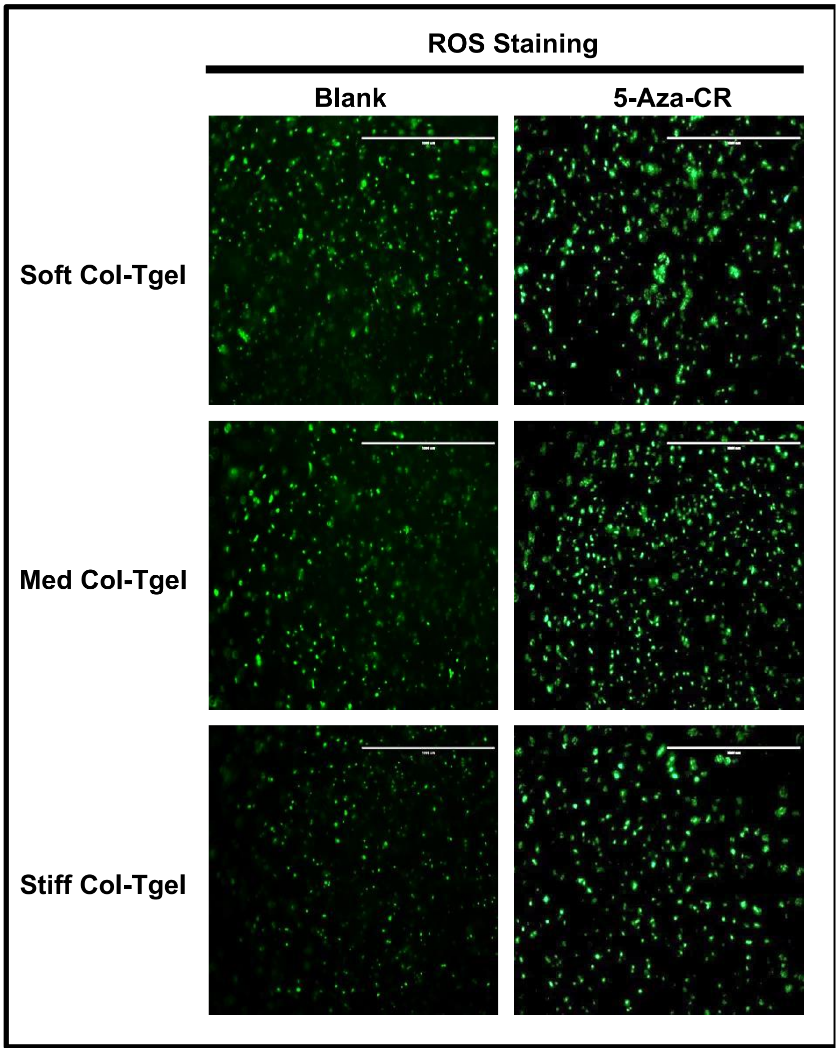

Fig. S4. **Endogenous cellular activation.** ADSCs were encapsulated in the Soft, Med, or Stiff Col-Tgel and grown with or without 5-Aza-CR. Reactive oxygen species (ROS) staining. 5-Aza-CR enhanced ROS production. Regardless of 5-Aza-CR, ROS production was highest in the softer matrices (Scale bars, 1000 μm).

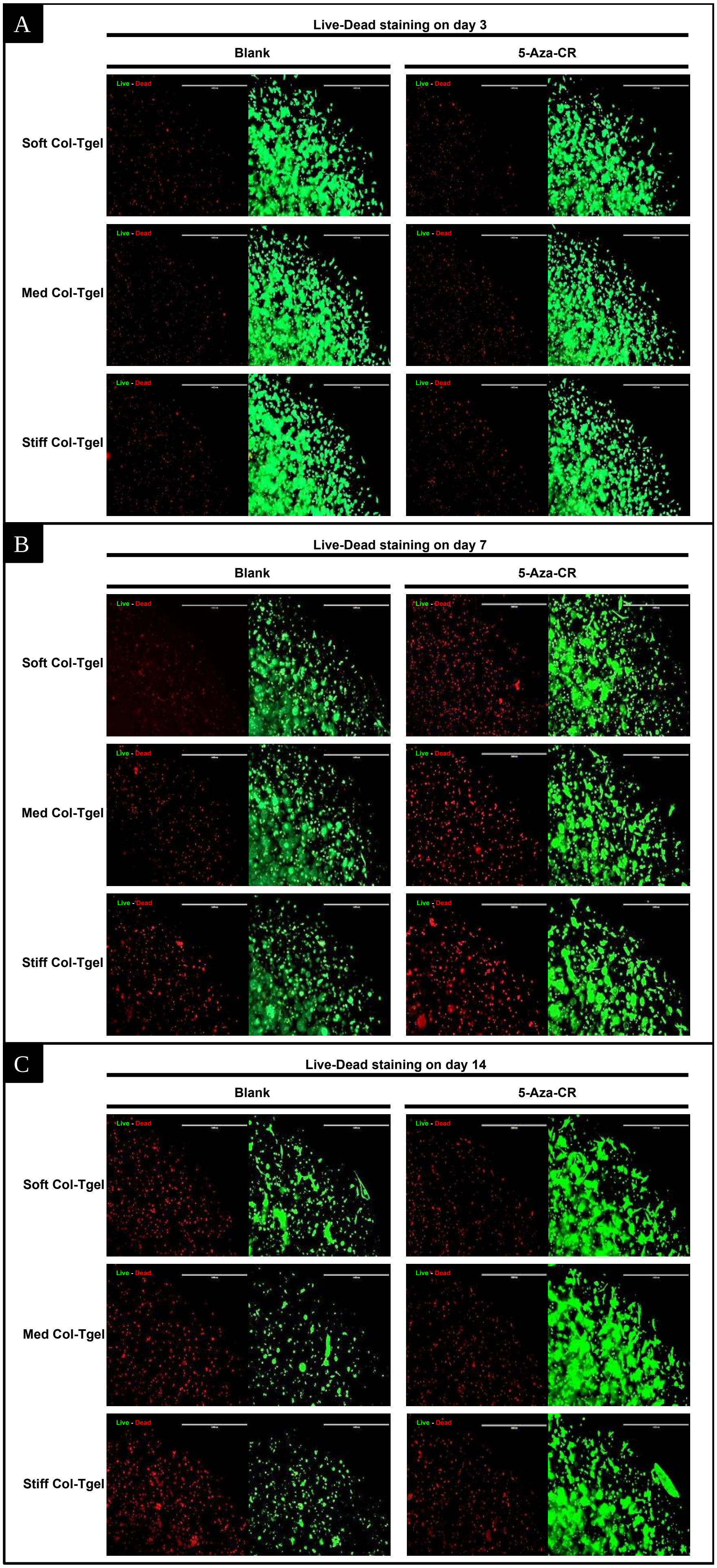
A
Live-Dead staining on day 3
Blank
5-Aza-CR
Soft Col-Tgel
Med Col-Tgel
Stiff Col-Tgel
B
Live-Dead staining on day 7
Blank
5-Aza-CR
Soft Col-Tgel
Med Col-Tgel
Stiff Col-Tgel
C
Live-Dead staining on day 14
Blank
5-Aza-CR
Soft Col-Tgel
Med Col-Tgel
Stiff Col-Tgel
Live - Dead

Fig. S5. **Cell viability and cytotoxicity.** ADSCs were encapsulated in the Soft, Med, or Stiff Col-Tgel and grown with or without 5-Aza-CR. (*A*), (*B*) and (*C*). Live/dead cell viability/cytotoxicity staining on day 3, 7 and 14, respectively. The staining showed that initially there were no significant differences between with and without the additional of 5-Aza-CR. Although the supplement of 5-Aza-CR caused more cell death on day 7, the live cells treated with 5-Aza-CR remained comparable to the cells that were treated without any treatment. Importantly, the ADSCs treated with 5-Aza-CR had higher survival rate than the ADSCs without any treatment, where the death cells were rejuvenated through proliferation on day 14 (Scale bars, 1000μm).

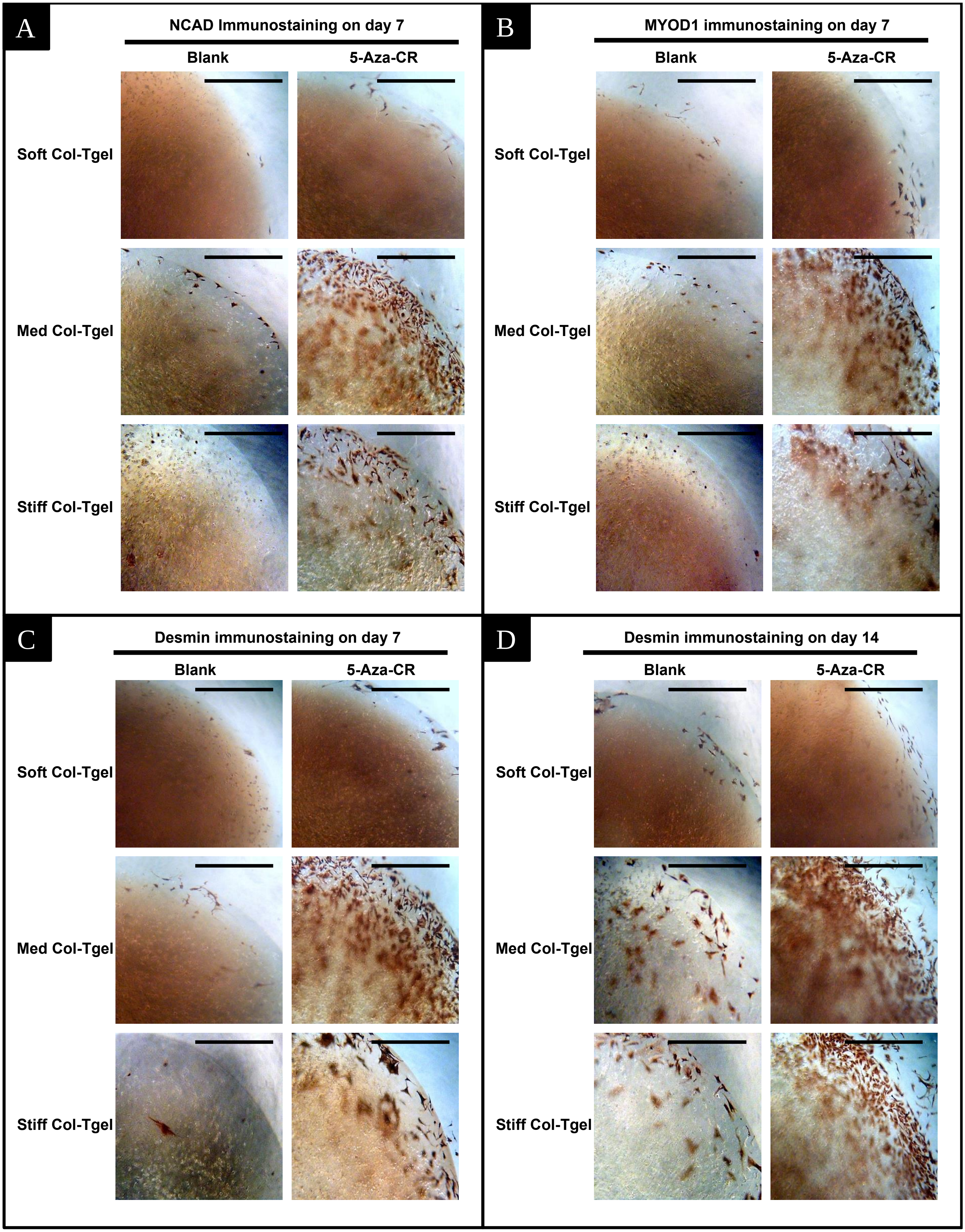

A
NCAD Immunostaining on day 7
Blank
5-Aza-CR
Soft Col-Tgel
Med Col-Tgel
Stiff Col-Tgel
B
MYOD1 immunostaining on day 7
Blank
5-Aza-CR
Soft Col-Tgel
Med Col-Tgel
Stiff Col-Tgel
C
Desmin immunostaining on day 7
Blank
5-Aza-CR
Soft Col-Tgel
Med Col-Tgel
Stiff Col-Tgel
D
Desmin immunostaining on day 14
Blank
5-Aza-CR
Soft Col-Tgel
Med Col-Tgel
Stiff Col-Tgel

Fig. S6. **Reprogramming ADSCs into myoblast-like cells.** ADSCs were encapsulated in the Soft, Med, or Stiff Col-Tgel and grown with or without 5-Aza-CR. (*A*) and (*B*) CDH2 (N-Cadherin) and MYOD1 on day 7, respectively. (*C*) and (*D*) DES (Desmin) on day 7 and 14, respectively. The supplement of 5-Aza-CR significantly up-regulated the expression of CDH2, MYOD1 and DES on day 7 regardless of gel conditions. The myogenic markers were obviously enhanced in the Med Col-Tgel and the least amount of myogenic markers was found in the Soft Col-Tgel with and without the addition of 5-aza-CR on day 7. On day 14, myoblasts-like cells were gradually expressed by ADSCs that were treated with 5-aza-CR. Likewise, the trans-differentiation of ADSCs into myoblasts-cells were lavishly expressed in the Med Col-Tgel and only limited myoblasts-cells were found in the Soft Col-Tgel regardless of the addition of 5-Aza-CR (Scale bars, 1000μm).

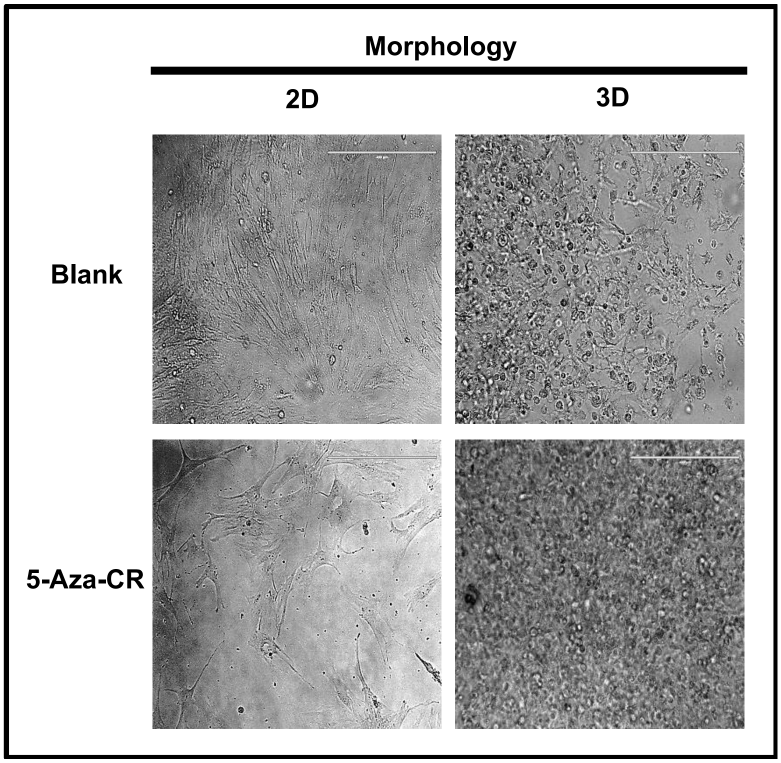

Fig. S7. **Cell morphology of hADSCs.** hADSCs were cultured on 2D surface or encapsulated in Col-Tgel. hADSCs on 2D surface displays branching morphology while cells become less elongated when were encapsulated in 3D Col-Tgel (Scale bars, 400μm).

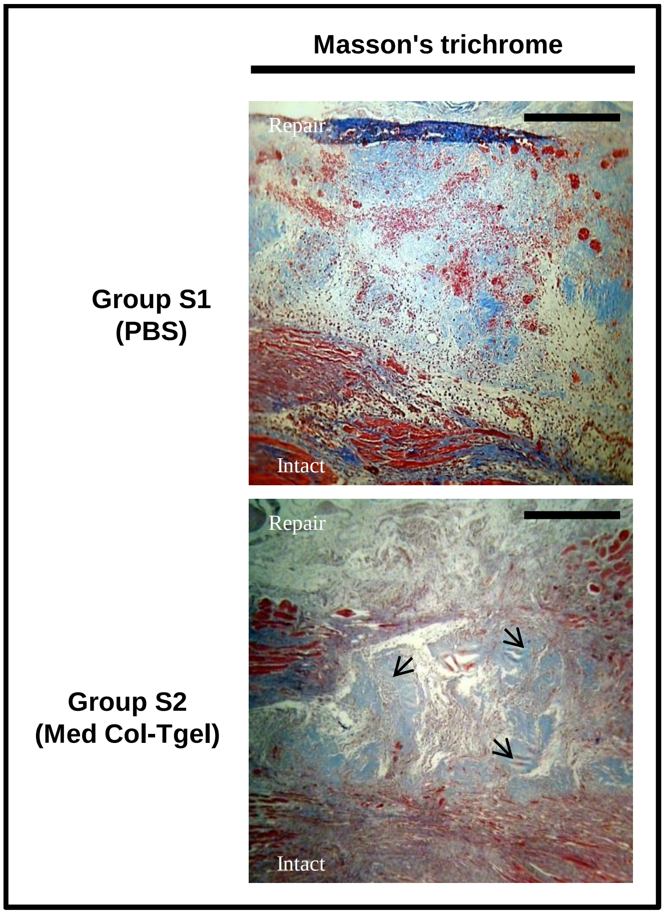

Fig. S8. ***In vivo* tissue regeneration.** Histology evaluation after 14 days of treatment. Significant fibrosis and hemorrhage were found in Group S1. Although Group S2 prevents fibrosis, least muscle infiltration was observed in Group S2. Black arrows indicate gel (Scale bars, 1000 μm).

SI Table

Table S1. List of antibodies and working dilutions used for immunostaining analysis.

Primary Antibody	Antigen	Isotype of Primary Antibody	Manufacturer of Primary Antibody	Dilution Factor for Immunostaining
OCT4	POU5F1 (POU domain, class 5, transcription factor 1)	Rabbit	Abcam, Cambridge, MA	1:200
ABCG2	ATP-binding cassette sub-family G member 2	Goat	Santa Cruz Biotechnology, Santa Cruz, CA	1:100
HIF-1α	Hypoxia-inducible factor 1-alpha	Rabbit	Bethyl Laboratories, Montgomery, TX	1:200
β1-integrins	Beta-1-integrins	Rabbit	R&D System, Minneapolis, MN	1:400
VEGF	Vascular endothelial growth factor	Rabbit	R&D System, Minneapolis, MN	1:400
Ki-67	Ki-67	Rabbit	R&D System, Minneapolis, MN	1:400
NCAD	N-Cadherin	Rabbit	R&D System, Minneapolis, MN	1:400
MYOD1	Myogenic determination factor 1	Rabbit	Abcam, Cambridge, MA	1:400
DES	Desmin	Rabbit	R&D System, Minneapolis, MN	1:400
DES	Desmin	Goat	R&D System, Minneapolis, MN	1:400

Table S2. Primers used to amplify mRNAs encoding mouse GAPDH.

**Primer**	**Sequence (5′–3′)**
LPL – F	AACAAGGTCAGAGCCAAGAG
LPL – R	CCATCCTCAGTCCCAGAAAAG
POU5F1 (OCT4) – F	AGTGGAAAGCAACTCAGAGG
POU5F1 (OCT4) – R	AACTGTTCTAGCTCCTTCTGC
ABCG2 – F	CAGGGTCATTCAAGAGTTAGGTC
ABCG2 – R	AGAACAAGATGGAAGGATCAGTG
HIF-1α – F	AACATAAAGTCTGCAACATGGAAG
HIF-1α – R	TTTGATGGGTGAGGAATGGG
MYOD – F	CCAATGCGATTTATCAGGTGC
MYOD – R	CGAAAGGACAGTTGGGAAGAG
Myogenin – F	CTGCCTAAAGTGGAGATCCTG
Myogenin – R	TGGGAGTTGCATTCACTGG
GAPDH – F	ATGGGGAAGGTGAAGGTCG
GAPDH – R	TAAAAGCATCCCTGGTGACC
